# The Role of Personal Values in the Context of the Relationship Between Perceived Stress and Satisfaction with Life in the Group of Uniformed Personnel Treated in a Mental Health Clinic

**DOI:** 10.3390/jcm15010369

**Published:** 2026-01-04

**Authors:** Mateusz Curyło, Michał Zabojszcz, Lidia Tkaczyk, Jaromira Iwolska, Marcin Mikos, Łukasz Strzępek, Aleksandra Czerw, Dorota Charkiewicz, Olga Partyka, Monika Pajewska, Katarzyna Sygit, Marian Sygit, Sławomir Wysocki, Izabela Gąska, Elżbieta Kaczmar, Elżbieta Grochans, Anna M. Cybulska, Daria Schneider-Matyka, Ewa Bandurska, Weronika Ciećko, Jarosław Drobnik, Piotr Pobrotyn, Dorota Waśko-Czopnik, Tomasz Sowiński, Julia Pobrotyn, Adam Wiatkowski, Tomasz Czapla, Monika Borzuchowska, Remigiusz Kozlowski

**Affiliations:** 1 Institute of Health Sciences, University of the National Education Commission, 30-084 Krakow, Poland; 2 Psychotherapy Day-Care Unit, Hospital of the Ministry of Interior and Administration in Krakow, 30-053 Krakow, Poland; 3 Institute of Medical Sciences, Collegium Medicum, Jan Kochanowski University, 25-317 Kielce, Poland; 4 Department of Bioinformatics and Public Health, Andrzej Frycz Modrzewski Krakow University, 30-705 Krakow, Poland; 5 Department of Surgery, Andrzej Frycz Modrzewski Cracow University, 30-705 Krakow, Poland; 6 Clinical Department of General and Oncological Surgery, Saint Raphael Hospital, 30-693 Krakow, Poland; 7 Department of Health Economics and Insurance, Medical University of Warsaw, 00-581 Warsaw, Poland; 8 Department of Economic and System Analyses, National Institute of Public Health NIH-National Research Institute, 00-791 Warsaw, Poland; 9 Faculty of Medicine and Health Sciences, University of Kalisz, 62-800 Kalisz, Poland; 10 Provincial Specialised Healthcare Complex for Lung Diseases and Tuberculosis in Wolica, 62-872 Wolica, Poland; 11 Medical Institute, Jan Grodek State University in Sanok, 38-500 Sanok, Poland; 12 Department of Nursing, Faculty of Health Sciences, Pomeranian Medical University in Szczecin, 71-210 Szczecin, Poland; 13 Center for Competence Development, Integrated Care and e-Health, Medical University of Gdansk, 80-204 Gdansk, Poland; 14 Department of Family Medicine, Faculty of Medicine, Wroclaw Medical University, 51-141 Wroclaw, Poland; 15 Citodent Dental Center, Furtak-Pobrotyn & Company Limited Partnership, 05-220 Olawa, Poland; 16 Department of Gastroenterology, Hepatology with Inflammatory Bowel Disease Subunit, Provincial Specialist Hospital J. Gromkowskiego, 51-149 Wroclaw, Poland; 17 Department of Non-Surgical Clinical Sciences, Faculty of Medicine, Wrocław University of Science and Technology, 50-370 Wrocław, Poland; 18 Endocare Medical Center, Simple Joint-Stock Company (S.J.S.C.), 50-558 Wroclaw, Poland; 19 Faculty of Medicine, Wroclaw Medical University, 50-345 Wroclaw, Poland; 20 Department of Management, Faculty of Management, University of Lodz, 90-237 Lodz, Poland; 21 Department of Management and Logistics in Healthcare, Medical University of Lodz, 90-131 Lodz, Poland

**Keywords:** stress, satisfaction with life, personal values, uniformed personnel

## Abstract

**Background/Objectives**: Personal values shape appraisal of stress and life satisfaction. We examined the relationship between perceived stress and life satisfaction among uniformed personnel in outpatient mental health care, and the role of a personal values hierarchy in this context. **Methods**: Cross-sectional study of 183 uniformed personnel (34 females, 149 males, age 30–66 years, M = 44.72, SD = 5.84) diagnosed with bodily distress disorder or post-traumatic stress disorder at a mental health clinic. Participants completed standardized questionnaires assessing perceived stress, satisfaction with life, coping styles, and personal values. For the Personal Value List, each value not selected by a participant was coded as 0 to avoid missing data; scores regarding symbols of happiness were not used. Reliability was evaluated via repeated measurement; two parts of a key instrument showed test–retest correlations of approximately 0.78 and 0.76. For assessing statistical significance, the bootstrap method was used (1000 resamples). Analyses were conducted in jamovi 2.3.28 using snowLatent (latent profile analysis) and medmod 1.1.0 (moderation analysis). **Results**: Perceived stress was negatively associated with satisfaction with life (B = −0.36, 95% CI [−0.48; −0.24], *p* < 0.001). Latent profile analysis extracted two personal values hierarchy profiles (AIC = 4237; BIC = 4587). Profile membership was not a significant predictor of satisfaction with life (*p* = 0.595) and did not moderate the relationship between perceived stress and satisfaction with life (*p* = 0.907). Distraction seeking was significantly higher in profile 1 (*p* = 0.010). **Conclusions**: In treated uniformed personnel, higher perceived stress is linked to lower life satisfaction. The personal values hierarchy did not moderate this relationship and was not associated with satisfaction with life; however, the personal values hierarchy was related to coping, specifically distraction seeking.

## 1. Introduction

Values are a set of personal needs, endeavors, desires, and aspirations forming an organization of beliefs regarding preferable ways of behaving, proceeding, but also final states of existence [1]. This organization implies a continuum of relative importance. The relative importance of values has a regulatory effect on one’s behavior, cognition, and affects and shapes attitudes. The higher the rank of a value in a hierarchy, the greater the impact on the functioning of an individual and his or her perception.

Based on data, stress can be defined as a disruption of homeostatic balance, affecting both physiological and psychological domains [2]. A high level of stress arises when the body and mind are subjected to excessive demands. Prolonged or chronic stress may lead to significant adverse outcomes. Perceived stress represents a cognitive construct reflecting an individual’s subjective appraisal of stress intensity [3]. The same situation may be evaluated as more or less stressful depending on the individual’s perception of its predictability, controllability, or degree of overload.

Perceived stress refers to an individual’s subjective experience because the evaluation is made against a personal benchmark, shaped by one’s perceived abilities and personal value hierarchy. Although health, energy, and prosperity may be desirable for many individuals, people differ in the importance assigned to these values. Therefore, depending on the values hierarchy, a stressful situation may be assessed as lowering or not lowering satisfaction with life, and different coping styles may be perceived as appropriate.

Life satisfaction refers to a cognitive and judgmental process through which individuals evaluate the overall quality of their lives [4]. It is a construct grounded in subjective perception and personal appraisal. Higher levels of perceived stress have been found to co-occur with, or predict, lower levels of life satisfaction [5].

Coping is defined as an individual’s attempt to manage distressing problems and emotions that shape the physical and psychological outcomes of stress [2]. It includes a range of behavioral and cognitive strategies that mediate the link between stressors and their effects.

A coping style refers to a characteristic pattern of responding to and managing stressful situations specific to an individual [6]. According to Endler [7], three primary coping styles can be distinguished: task-oriented, emotion-oriented, and avoidance-oriented. Task-oriented coping involves efforts directed toward solving the problem or altering the stressful situation itself. Emotion-oriented coping focuses on managing and processing the emotional responses associated with stress. Avoidance-oriented coping consists of behaviors and cognitions aimed at evading the stressful situation, which may take the form of engaging in distracting activities or seeking social diversion. Endler describes task-oriented coping as the most effective approach in situations where a degree of control can be exercised. Emotion-oriented coping is considered more adaptive when a situation is uncontrollable, while avoidance-oriented coping may serve as a temporary response to stress, though its effectiveness tends to diminish over time. Overall, task-oriented coping is regarded as the most effective strategy in the long-term perspective.

However, the course of action of an individual in a stressful situation can also be associated with their personal values hierarchy. Therefore, we formulated the following hypotheses:

**H1.**  *The level of perceived stress is associated negatively with satisfaction with life.*

**H2.**  *Personal values hierarchy is related to satisfaction with life.*

**H3.**  *Personal values hierarchy is related to coping with stress.*

We also formulated a research question to examine whether the personal values hierarchy is a moderator of the relationship between the level of perceived stress and satisfaction with life.

We tested these hypotheses using data collected from a specific sample of uniformed personnel diagnosed with either bodily distress disorder (6C20) or post-traumatic stress disorder (6B40). Uniformed services constitute a distinct occupational group, as their professional duties often entail exposure to both primary and secondary trauma [8]. Even routine tasks in this field require the use of specific coping strategies [9]. Consequently, dedicated psychological models have been developed to train and enhance appropriate coping mechanisms within this professional population [10,11].

Due to their highly stressful work environment, uniformed personnel face an elevated risk of developing mental disorders [12]. A study of U.S. Army soldiers found that 25.1% of participants met criteria for at least one disorder, and 11.1% met criteria for multiple disorders [13].

One potential consequence of chronic stress linked to high-demand professional work is the development of bodily distress disorder (6C20). This condition is marked by distressing physical symptoms that draw excessive attention to their nature and course over time. The symptoms typically occur on most days and persist for at least several months. Individuals may experience multiple bodily symptoms that can fluctuate over time, although a single persistent symptom, most commonly pain or fatigue, may also be present. Both the symptoms themselves and the associated distress and hypervigilance exert a substantial impact on an individual’s daily functioning and overall well-being. ICD-11 distinguishes three types of the disorder, depending on severity, i.e., mild, moderate, and severe. Bodily distress disorder needs to be differentiated with mood disorders, generalized anxiety disorder, panic disorder, health anxiety disorder, and factitious disorder imposed on self.

Another mental disorder common among uniformed personnel is post-traumatic stress disorder (6B40), which can develop after exposure to extremely threatening or horrific events [14]. PTSD most commonly involves re-experiencing the traumatic event through vivid, intrusive memories, flashbacks, or nightmares accompanied by intense emotions and, at times, physical sensations. Another symptom cluster is avoidance of trauma-related thoughts and memories as well as activities, situations, or people that may serve as reminders. In addition, individuals often experience persistent perceptions of heightened current threat. For a PTSD diagnosis, symptoms must persist for at least several weeks and lead to clinically significant impairment in functioning.

## 2. Materials and Methods

### 2.1. Study Design and Setting

This was a cross-sectional study conducted in the day psychotherapy ward at the Hospital of the Ministry of Interior and Administration in Kraków, Poland.

### 2.2. Participants

The group examined in the current study consisted of 183 uniformed personnel members, 34 females and 149 males aged 30–66 (M = 44.72; SD = 5.84). They were treated at a mental health clinic and received outpatient care from February 2024 to August 2024. Participating patients were diagnosed with bodily distress disorder (6C20) or post-traumatic stress disorder (6B40) [15]. No additional eligibility criteria were applied. The therapy lasted 5–6 weeks. Of the 193 patients who met the inclusion criteria, 10 (5.2%) declined to participate. Table 1 presents sociodemographic characteristics of the group examined in the current study. It includes distributions of gender and the level of education, and means, standard deviations, and minimum and maximum values regarding the age of participants, depending on gender and the level of education.

Most participants were male, and most had completed higher education. Participants’ age did not differ across groups defined by gender or educational level.

### 2.3. Procedure and Ethics

Participants filled out, at the same time, psychological questionnaires. The order of questionnaires was as follows: CISS, SWLS, PSS-10, and Juczynski’ Personal Value List.

The questionnaires were administered at the Hospital of the Ministry of Interior and Administration in Kraków, in the day psychotherapy ward where the study was conducted. Data were collected by a qualified psychologist employed at the facility. The study received approval from the bioethics committee, permission from the hospital director to conduct the research on site, and written informed consent from participants was obtained by the psychologist. The consent forms, together with the completed questionnaires, are stored in the hospital’s archives.

### 2.4. Measures

Coping Inventory for Stressful Situations (CISS), originally developed by Endler and Parker [16] and adapted as a Polish version [17], was used to assess stress coping styles. The questionnaire consists of 48 diagnostic items measuring three types of coping: emotion-oriented, task-oriented, and avoidant. The avoidant style is divided into two subscales: distraction and social diversion.

The Perceived Stress Scale (PSS-10) [18] was applied to assess the level of perceived stress intensity. The instrument consists of ten items rated on a 5-point scale ranging from 0 to 4. Four items are reverse-scored before computing the total result. The overall score may range from 0 to 40 points, with higher scores reflecting greater levels of perceived stress.

The Satisfaction with Life Scale (SWLS) [18] was administered to evaluate life satisfaction. This questionnaire comprises five items rated on a 7-point Likert scale, where higher values indicate greater agreement with the statements. The total score ranges from 5 to 35 points, with higher results corresponding to a higher level of life satisfaction.

Juczynski’s Personal Value List [18] was used to assess the personal values. The questionnaire uses a hierarchical approach and it consists of two parts. The first part lists nine so-called symbols of happiness expressing various forms of human values actualization, like a large circle of friends, good financial conditions, or fame and popularity. The second part lists ten categories of personal values including good health, but also sense of humor, wealth and possessions, or knowledge and wisdom. The questionnaires measure the importance ascribed to the symbols of happiness and values. Out of nine symbols of happiness, each participant chooses five and then gives them scores ranging from 5—the most important symbol, to 1—the least important symbol. The personal values are assessed the same way. As a result, a hierarchy of both happiness symbols and personal values can be acquired both in terms of the mean position of each symbol and value and in terms of a symbol and a value being chosen or not. However, for the purpose of the current study, we coded each value that was not indicated and chosen by a participant with the value of zero. This way, we avoided missing values, which would seriously limit further analyses. Also, we did not use the scores regarding the symbols of happiness and focused on the values. The reliability of the questionnaire was verified with the use of repeated measurements. The correlation coefficients between the two consecutive measurements were equal to 0.78 for the first part of the questionnaire and 0.76 for the second part if the second measurement was performed after two days, or 0.72 for the second part if the second measurement was performed after six weeks.

Indicators of the personal values hierarchy were further examined as potential predictors of life satisfaction and as potential moderators of the association between perceived stress and life satisfaction. To this end, we first conducted a latent profile analysis to identify subgroups of participants differing in their values hierarchies, and then performed a moderation analysis in which subgroup membership was tested as the moderator.

### 2.5. Statistical Analysis

For assessing the statistical significance, the bootstrap method was used. With the bootstrap method, we drew 1000 subsamples from the current sample with replacement and calculated regression coefficients each time a subsample was drawn. This approach let us perform parametric statistical analysis without assuming the residuals to be normally distributed.

We used jamovi 2.3.28 software [19] with the additional modules snowLatent for the purpose of latent profile analysis and medmod 1.1.0., devoloped for the purpose of moderation and mediation analysis. Also, we analyzed the differences between extracted subgroups in terms of stress coping styles with MANOVA.

## 3. Results

### 3.1. Descriptive Statistics

Table 2 presents the descriptive statistics for the analyzed interval variables, including mean values, standard deviations, minimum and maximum scores, as well as measures of skewness and kurtosis. The Shapiro–Wilk test values for normality are also reported, along with the Cronbach’s alpha coefficients, indicating the reliability of the applied scales.

Internal consistency (Cronbach’s α) for the analyzed scales was acceptable to high in this sample. Specifically, α equaled 0.88 for task-oriented coping, 0.91 for emotion-oriented coping, 0.83 for avoidant coping, 0.76 for distraction seeking, and 0.75 for social diversion. For the well-being variables, α equaled 0.88 for perceived stress (PSS-10) and 0.76 for satisfaction with life (SWLS).

The Shapiro–Wilk tests indicated significant deviations from normality for task-oriented coping (W = 0.87, *p* < 0.001), emotion-oriented coping (S-W = 0.98, *p* = 0.031), avoidant coping (W = 0.97, *p* < 0.001), and social diversion (S-W = 0.96, *p* < 0.001). All personal values indicators also deviated from normality (all *p* < 0.001). In contrast, perceived stress (S-W = 0.99, *p* = 0.293), satisfaction with life (S-W = 0.99, *p* = 0.065), and distraction seeking (S-W = 0.99, *p* = 0.073) did not show statistically significant departures from normality at α = 0.05.

In this sample, the mean perceived stress score was 19.30 (SD = 6.38; range 5–37), and the mean satisfaction with life score was 21.00 (SD = 5.49; range 5–33). Mean coping scores were task-oriented 53.60 (SD = 11.26), emotion-oriented 42.60 (SD = 11.47), avoidant 42.10 (SD = 9.89), distraction seeking 19.30 (SD = 5.13), and social diversion 14.80 (SD = 4.25).

The distributions of task-oriented, emotion-oriented, and avoidant coping, as well as social diversion, and all indicators of personal values, deviated significantly from normality.

### 3.2. Latent Profile Analysis

**Figure 1:**
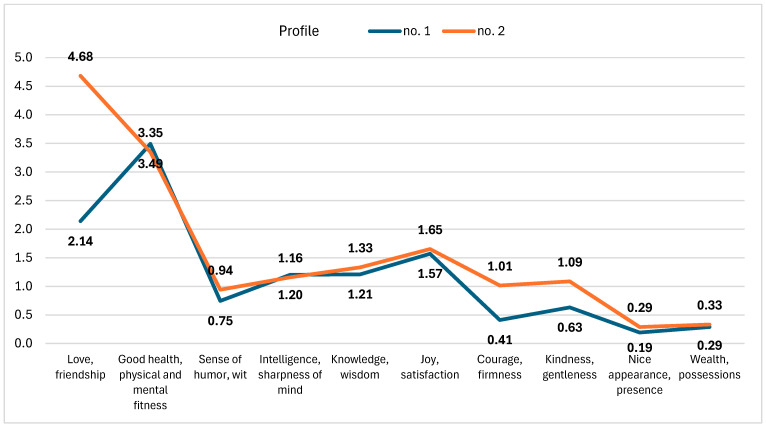
Profiles of personal values hierarchy extracted in the course of the latent profile analysis.

In the course of latent profile analysis, two subgroups with distinctive profiles were extracted. The fit indices were equal to *AIC* = 4237 and *BIC* = 4587, respectively. Figure 1 depicts the profiles acquired.

Two profiles were identified: profile no. 1 (*n* = 114) and profile no. 2 (*n* = 69). The profiles differed primarily in the relative importance of love and friendship versus good health, and physical and mental fitness. In profile no. 1, mean scores for love and friendship and for good health were 2.14 and 3.49, respectively, whereas in profile no. 2, the corresponding means were 4.68 and 3.35. Differences were also observed for courage and firmness (0.41 in profile no. 1 to 1.01 in profile no. 2) and kindness and gentleness (0.63 to 1.09). The remaining value indicators showed smaller differences between profiles (e.g., joy, satisfaction—1.57 to 1.65; wealth, possessions—0.29 to 0.33).

### 3.3. Moderation Analysis

Personal values profile subgroup membership was examined both as a predictor of life satisfaction and as a moderator of the association between perceived stress and life satisfaction. Table 3 presents regression coefficients acquired in the moderation analysis performed.

In the regression model, perceived stress was a significant negative predictor of satisfaction with life (B = −0.36, 95% CI [−0.48; −0.24], Z = −5.68, *p* < 0.001), indicating that higher perceived stress corresponded to lower life satisfaction, supporting hypothesis H1. Profile membership alone was not a significant predictor (B = 0.44, 95% CI [−1.22; 2.07], Z = 0.53, *p* = 0.595). The interaction term representing the moderation effect was also non-significant (B = 0.01, 95% CI [−0.26; 0.26], Z = 0.12, *p* = 0.907), indicating that the strength of the stress–life satisfaction association did not differ between the two personal values profiles; therefore, hypothesis H2 was not confirmed.

### 3.4. Personal Values Hierarchy and Stress Coping Styles

We compared the two subgroups extracted with latent profile analysis using MANOVA. Test was close to statistical significance, F(5, 176) = 1.98, *p* = *0*.083. Table 4 presents the mean values for each stress coping style with the values of ANOVA.

Although the multivariate test approached statistical significance (F(5, 176) = 1.98, *p* = 0.083), the univariate comparisons clarified that the profiles differed specifically in distraction seeking. Participants in profile no. 1 showed higher distraction seeking (M = 20.00, SD = 4.73) than participants in profile no. 2 (M = 18.00, SD = 5.53), with F(1, 180) = 6.75, *p* = 0.010. No statistically significant differences were observed for task-oriented coping (*p* = 0.918), emotion-oriented coping (*p* = 0.822), overall avoidant coping (*p* = 0.105), or social diversion (*p* = 0.980). These results were consistent with Hypothesis H3.

## 4. Discussion

Our findings confirmed a negative association between perceived stress and life satisfaction. Moreover, this relationship was not moderated by the personal hierarchy of symbols of happiness or the hierarchy of personal values, which suggests that the relationship between perceived stress and satisfaction with life is deeply seated. It is not reinforced or modified by a personal values hierarchy. However, perceived stress and satisfaction with life are not tautological concepts. Measuring perceived stress involves the perception regarding the last month, while measuring satisfaction with life involves the overall perspective on one’s life in general.

The personal hierarchy of personal values was not related to satisfaction with life. This result is not consistent with some other studies. For example, in the study conducted on a sample of nurses [20], which is also a profession associated with a high level of occupational stress, it was found that identification with self-transcendence values was related to nurses’ life satisfaction. Therefore, values, like concern for others, care for people in general, understanding, and tolerance as the opposite of a materialistic and individual approach, are associated with better life satisfaction.

Regarding the analysis of relationships between personal values, and coping with stress, the importance of love and friendship, courage and firmness and kindness and gentleness were found to be negatively related to distraction seeking. It may be that these values facilitate social relationships, which if established and profound, do not need avoidance in distraction seeking to be involved in coping with stress.

In other recent research, personal values were analyzed in the context of coping with the COVID-19 pandemic. It was found that prioritizing communal values as opposed to agentic values was associated with coping with prevention behaviors to reduce virus transmission, and also with motivations to help others suffering from the pandemic [21]. Another study was focused on the differences between females and males [22]. The main conclusion was that values that were valued mostly by females were one of the factors contributing to their level of anxiety and depression and the values important for male students were helpful in coping with stressful situations, which explained the lower level of both anxiety and depression in the group of male participants. In another study [23], simultaneous contributions of the coping strategies and individual values were used to predict the level of psychological resilience.

Certainly, our current study has several limitations. Firstly, it was conducted on a specific sample, which limits the generalization of our findings. Moreover, it was a cross-sectional study. A longitudinal study would allow for examining how potential changes in the personal values of hierarchy, for example, in the course of treatment, affect coping and satisfaction with life. In the current study, we did not measure symptom severity; therefore, no additional analysis regarding relationships between personal values, the level of stress, and quality of life was possible. Numbers of males and females were disproportionate, and males and females differed regarding the level of stress and satisfaction with life. Finally, a comparison with results acquired on a similar sample of participants, but without clinical symptoms currently elevated, would allow for additional valuable results.

## 5. Conclusions

The findings confirmed a significant negative association between perceived stress and life satisfaction in uniformed personnel diagnosed with bodily distress disorder or post-traumatic stress disorder. The hierarchy of personal values did not moderate this relationship and was not associated with life satisfaction. The hierarchy of personal values was significantly related with stress coping, specifically with distraction seeking.

Based on the results, implications for clinical practice were prepared:

1. Implement interventions, such as Acceptance and Commitment Therapy (ACT) or Motivational Interviewing, to align coping strategies with adaptive values, as personal values are related to coping;
2. Use follow-up assessments of value hierarchies and coping styles to guide ongoing treatment and detect early warning signs of maladaptive coping, as perceived stress is related to satisfaction with life;
3. Provide mental health professionals with training in value-oriented approaches to strengthen therapeutic alliance and enhance treatment outcomes, as personal values are related to stress coping.

Further longitudinal and comparative studies are recommended to examine causal relationships and to determine whether value-based interventions can sustainably improve coping strategies and overall life satisfaction in uniformed personnel. Research including non-clinical control groups would help clarify whether the observed patterns are unique to individuals with clinical diagnoses.

## Figures and Tables

**Table 1:** Sociodemographic characteristics of the group in the current sample.

	Gender		Education	
	Female	Male	Secondary	Higher
*n*	34 (18.6%)	149 (81.4%)	89 (48.6%)	94 (51.4%)
M	46.94	44.21	45.55	44.17
SD	8.38	4.99	6.11	6.12
min	30	33	33	30
max	66	57	66	60

*n*—number of participants; M—mean value; SD—standard deviation; min—minimum value; max—maximum value.

**Table 2:** Descriptive statistics for stress coping styles, the level of perceived stress, and satisfaction with life.

Variables	M	SD	min	max	S	K	S-W	*p*	α
Stress coping styles									
Task-oriented style	53.60	11.26	13	75	−1.51	3.65	0.87	<0.001	0.88
Emotion-oriented style	42.60	11.47	10	66	−0.37	0.06	0.98	0.031	0.91
Avoidant style	42.10	9.89	16	62	−0.61	0.33	0.97	<0.0001	0.83
Distraction seeking	19.30	5.13	8	31	−0.10	−0.59	0.99	0.073	0.76
Social diversion	14.80	4.25	2	23	−0.72	0.50	0.96	<0.001	0.75
Perceived stress	19.30	6.38	5	37	−0.02	−0.30	0.99	0.293	0.88
Satisfaction with life	21.00	5.49	5	33	−0.19	−0.28	0.99	0.065	0.76
Personal values									
Love, friendship	3.10	2.02	0	5	−0.63	−1.32	0.77	<0.001	-
Good health, physical and mental fitness	3.44	1.86	0	5	−0.96	−0.61	0.76	<0.001	-
Sense of humor, wit	0.82	1.42	0	6	1.71	1.83	0.64	<0.001	-
Intelligence, sharpness of mind	1.19	1.64	0	10	1.47	3.33	0.72	<0.001	-
Knowledge, wisdom	1.26	1.44	0	5	0.73	−0.72	0.80	<0.001	-
Joy, satisfaction	1.60	1.57	0	8	0.67	0.14	0.85	<0.001	-
Courage, firmness	0.64	1.13	0	5	1.82	2.48	0.63	<0.001	-
Kindness, gentleness	0.80	1.34	0	6	1.63	1.75	0.66	<0.001	-
Nice appearance, presence	0.23	0.93	0	9	6.02	46.14	0.27	<0.001	-
Wealth, possessions	0.31	0.90	0	5	3.50	12.35	0.39	<0.001	-

M—mean value; SD—standard deviation; min—minimum value; max—maximum value; S—skewness; K—kurtosis; S-W—Shapiro–Wilk test for normality; *p*—statistical significance; α—Cronbach’s α reliability coefficient.

**Table 3:** Analysis of the personal values hierarchy in the role of predictors of satisfaction with life and moderators of the relationship between the level of perceived stress and satisfaction with life.

Effect	B	Z	*p*
Perceived stress	−0.36 [−0.48; −0.24]	−5.68	<0.001
Personal values—profile no. 1 or no. 2	0.44 [−1.22; 2.07]	0.53	0.595
Moderation effect	0.01 [−0.26; 0.26]	0.12	0.907

B—regression coefficient; Z—test for regression coefficient’s significance; *p*—statistical significance.

**Table 4:** Mean values for each stress coping style in the subgroups with distinctive profiles of personal values hierarchy.

	Profile of Personal Values						
	No. 1		No. 2				
Stress Coping Styles	M	SD	M	SD	F	df	*p*
Task-oriented style	53.70	9.88	53.50	13.30	0.01	1180	0.918
Emotion-oriented style	42.80	11.00	42.40	12.30	0.05	1180	0.822
Avoidant style	43.00	9.14	40.60	10.90	2.65	1180	0.105
Distraction seeking	20.00	4.73	18.00	5.53	6.75	1180	0.010
Social diversion	14.80	4.06	14.80	4.58	0.00	1180	0.980

M—mean value; SD—standard deviation; F—ANOVA test; df—degrees of freedom; *p*—statistical significance.

## Data Availability

Data available from authors.
